# Exploring the potential of photodynamic therapy in overcoming multidrug resistance: mechanisms, synergies, and clinical advancements in infectious diseases

**DOI:** 10.3389/fcimb.2025.1624036

**Published:** 2025-08-14

**Authors:** Ruchita Tanu, Anis Ahmad Chaudhary, Gagan Prakash, Nusrath Yasmeen, Mohamed A. M. Ali, Nadeem Raza, Pushpender K. Sharma, Akhilesh Kumar, Tejpal Yadav, Vikram Kumar

**Affiliations:** ^1^ Amity Institute of Biotechnology, Amity University Rajasthan, Jaipur, Rajasthan, India; ^2^ Department of Biology, College of Science, Imam Mohammad Ibn Saud Islamic University (IMSIU), Riyadh, Saudi Arabia; ^3^ Department of Chemistry, College of Science, Imam Mohammad Ibn Saud Islamic University (IMSIU), Riyadh, Saudi Arabia; ^4^ Amity Institute of Pharmacy, Amity University Rajasthan, Jaipur, Rajasthan, India

**Keywords:** multidrug resistance, photodynamic therapy, reactive oxygen species, antimicrobial resistance, biofilm disruption, light-based therapies

## Abstract

Multidrug resistance (MDR) in bacterial and fungal pathogens poses a growing global health crisis, rendering many conventional antimicrobial therapies ineffective. The rise of MDR strains complicates treatment, prolongs illness, increases healthcare costs, and contributes to higher mortality rates. Mechanisms driving MDR include enzymatic drug inactivation, target modification, efflux pump activity, decreased permeability, and biofilm formation—often fueled by horizontal gene transfer and selective pressure from antimicrobial overuse. In response to the urgent need for novel therapeutic strategies, photodynamic therapy (PDT) has emerged as a promising, non-traditional approach. PDT utilizes a photosensitizing agent, light of a specific wavelength, and oxygen to generate reactive oxygen species (ROS) that inflict oxidative damage on microbial or cancer cells. This mechanism circumvents conventional resistance pathways, offering targeted, minimally invasive, and effective treatment for infections and malignancies. PDT is particularly adept at penetrating biofilms and resistant microbial populations, thus broadening its clinical applicability. In addition to direct microbial eradication, PDT may stimulate immune responses and demonstrates a favorable safety profile compared to traditional antibiotics or chemotherapy. Furthermore, advances in Antimicrobial Blue Light (aBL) and next-generation photosensitizers enhance PDT’s effectiveness while minimizing resistance development. This review explores the biological mechanisms underlying MDR, the principles and evolution of PDT, and its synergistic potential in managing infectious diseases. By addressing critical gaps in antimicrobial therapy, PDT stands out as a transformative modality in the ongoing battle against drug-resistant pathogens.

## 1 Introduction

The World Health Organization (WHO) identifies antimicrobial resistance (AMR), including multidrug resistance (MDR), as one of the top ten threats to global public health. In 2019, bacterial AMR was directly responsible for an estimated 1.27 million deaths worldwide, with MDR strains playing a major role in this toll (World Health Organization, 2023). The U.S. Centers for Disease Control and Prevention (CDC) highlighted MDR organisms such as carbapenem-resistant Enterobacteriaceae and drug-resistant Candida auris as urgent threats in its 2019 AMR Threats Report, noting that these pathogens cause thousands of deaths each year in the United States alone (Centers for Disease Control and Prevention | CDC, 2025). Similarly, data from the European Centre for Disease Prevention and Control (ECDC) show rising rates of resistance to multiple antibiotic classes among pathogens like Escherichia coli, Klebsiella pneumoniae, and Pseudomonas aeruginosa across Europe. These findings emphasize the urgent need for innovative antimicrobial strategies to address MDR, particularly as the development of new antibiotics lags behind the pace of emerging resistance (Murray et al., 2022). Multidrug resistance (MDR) is a crucial and escalating risk factor for infections caused by bacteria and fungi. It describes how several antimicrobial drugs can be resisted by microbes, making traditional treatments useless. This process happens when microbes evolve defenses against medications that used to effectively kill them or stop their growth (Habboush and Guzman, 2025). As MDR strains proliferate, treatment choices get more complex, healthcare expenses rise, hospital stays lengthen, and morbidity and mortality rates sharply rise (Majumder et al., 2020). The bacterial and fungal pathogens that cause common illnesses like pneumonia, bloodstream infections, urinary tract infections, and invasive fungal diseases are among the many that exhibit multidrug resistance (MDR). The advent of MDR strains has broad ramifications, affecting global public health activities and patient outcomes (Bharadwaj et al., 2022; Tanwar et al., 2014).

Microorganisms utilize various strategies to develop MDR. These strategies can be generally classified as enzymatic inactivation, in which bacteria and fungi generate enzymes that either degrade or modify antimicrobial agents, thereby hindering their ability to bind to target sites (Chinemerem Nwobodo et al., 2022). Another significant mechanism is targeting modification, where microorganisms change the structure of the antimicrobial agent’s target molecule, thereby reducing or completely eliminating the binding of the drug (Uddin et al., 2021). Efflux pumps play a vital role as well; these protein complexes actively expel antimicrobial agents from the microbial cell, decreasing their intracellular concentration and preventing access to their targets (Alenazy, 2022). Reduced permeability also contributes, as alterations in the microbial cell membrane or cell wall can lessen the entry of antimicrobial agents into the cell, limiting their access to target sites (Ghai and Ghai, 2018). Additionally, many microorganisms can develop biofilms, which are intricate communities of cells embedded in a self-produced matrix, offering a physical barrier that shields microorganisms from antimicrobial agents and immune responses (Rather et al., 2021). These mechanisms can be acquired via horizontal gene transfer (such as plasmids and transposons) or can emerge through spontaneous mutations. The widespread use of antimicrobial agents exerts selective pressure that fosters the evolution and proliferation of MDR strains (Reygaert, 2018).

There are many difficulties in treating MDR infections. The inability of conventional antibiotic treatments to completely remove the infection frequently results in longer disease duration, a higher risk of complications, and a higher death rate. Serious adverse effects may arise from the necessity of using substitute antimicrobial agents, which are frequently more toxic (Salam et al., 2023). Delays in diagnosing MDR strains can also make it more difficult to administer the right care, which can impair patient outcomes. Furthermore, the urgent need for innovative treatment approaches is highlighted by the limited supply of new antimicrobial medicines to address rising MDR strains. To tackle the problem of MDR infections, tactics like infection control procedures, antimicrobial stewardship programs, and the creation of novel medications and alternative treatments are essential (Ahmed et al., 2024; Huemer et al., 2020).

Photodynamic therapy (PDT) is a treatment method that employs a photosensitizing agent, light, and oxygen to provoke cell death and disrupt tissue. The foundation of PDT lies in the administration of a photosensitizer, an inert molecule that selectively concentrates in the target area, such as cancer cells or infected tissues. Following this, the tissue is subjected to light of a specific wavelength that aligns with the absorption characteristics of the photosensitizer. Once the light activates it, the photosensitizer undergoes a photochemical process, transferring energy to adjacent oxygen molecules and producing reactive oxygen species (ROS), including singlet oxygen. These ROS are extremely toxic to cells and inflict oxidative damage to various cellular structures, resulting in cell death via apoptosis, necrosis, or autophagy. The focused nature of PDT reduces harm to nearby healthy tissues, making it a promising option for the treatment of various medical conditions (Aebisher et al., 2024; Cieplik et al., 2013).

The origins of PDT date back to the early 1900s when scientists noticed the harmful effects of particular dyes when exposed to light (Fernández-Guarino et al., 2010). In 1900, Oscar Raab found that acridine dyes could be lethal to paramecia when activated by light. Subsequently, in 1903, Niels Finsen was awarded the Nobel Prize in Physiology or Medicine for his innovative use of light to treat skin tuberculosis (Szeimies et al., 2001). However, the contemporary understanding of PDT began to take shape in the 1970s, primarily due to the contributions of Thomas Dougherty, who illustrated the effectiveness of hematoporphyrin derivative (HpD) in fighting experimental tumors (Aebisher et al., 2024). This early photosensitizer, HpD, demonstrated a tendency to concentrate in tumor cells and was successful in promoting tumor shrinkage when exposed to light. Since then, PDT has seen considerable progress, leading to the creation of more potent photosensitizers, enhanced light delivery mechanisms, and refined treatment strategies (Miranda et al., 2018). Today, PDT is utilized clinically to address various ailments, including certain cancer types, skin disorders, and infections. Ongoing studies are continuously investigating new applications for PDT, aiming to improve its effectiveness and safety (Garcia de Miranda et al., 2018). Among the clinically approved protocols, 5-aminolevulinic acid (5-ALA)-based photodynamic therapy (PDT) has become a gold standard, especially for treating dermatological conditions such as actinic keratosis, superficial basal cell carcinoma, and acne. 5-ALA is a prodrug that, when administered, leads to the intracellular accumulation of protoporphyrin IX (PpIX), a powerful endogenous photosensitizer. Once activated by red or blue light, PpIX generates cytotoxic reactive oxygen species. The approval of 5-ALA and its methyl ester derivative, methyl aminolevulinate (MAL), has significantly facilitated the widespread adoption of PDT in clinical dermatology, showcasing high selectivity, minimal invasiveness, and favorable cosmetic outcomes (Gholami et al., 2023; Fernández-Guarino et al., 2010; Guleng and Helsing, 2012).

PDT is emerging as a promising approach for tackling MDR in microbial infections and cancer due to its distinctive mode of action. In contrast to conventional antimicrobial or chemotherapeutic drugs that typically target specific cellular sites, PDT triggers cell death by generating ROS, which inflict extensive oxidative damage on various cellular components. This non-targeted assault complicates the ability of microorganisms or cancer cells to establish resistance mechanisms against PDT (de Carvalho Leonel et al., 2019). Photodynamic therapy (PDT) can effectively overcome the physical and biochemical barriers posed by biofilms and tumor microenvironments by generating reactive oxygen species (ROS) on-site. Because ROS generation occurs locally where the photosensitizer has concentrated and is activated by light, PDT can target and damage both surface and embedded cells within these protective structures. This capability addresses a significant challenge in treating multidrug-resistant (MDR) infections and solid tumors. The capability of PDT to bypass standard resistance pathways makes it a significant asset in the fight against MDR infections and cancers that do not respond to traditional treatments (Liu et al., 2015; Youf et al., 2021).

PDT presents various potential benefits over standard treatment approaches for both infections and cancer. To begin with, PDT allows for highly targeted delivery to diseased tissues, thereby reducing harm to adjacent healthy tissues. This is possible through the application of photosensitizers that preferentially gather in the affected area, combined with accurate light transmission to activate the photosensitizer. In addition, PDT demonstrates a wide range of efficacy, capable of eliminating numerous microorganisms such as bacteria, fungi, and viruses, along with various types of cancer cells. Furthermore, PDT can trigger a strong immune response, potentially boost its therapeutic effects and help to prevent disease recurrence. It is also typically well-tolerated with fewer overall side effects in comparison to conventional chemotherapy or antibiotics. Moreover, the likelihood of developing resistance to PDT is low, rendering it a viable option for the ongoing management of multidrug-resistant infections and cancers. These benefits position PDT as an appealing alternative or complementary therapy to traditional treatments, especially in scenarios where multidrug resistance poses a significant challenge (González et al., 2021; Guleng and Helsing, 2012; Liu et al., 2015; Pérez-Laguna et al., 2021a).

**Figure 1:**
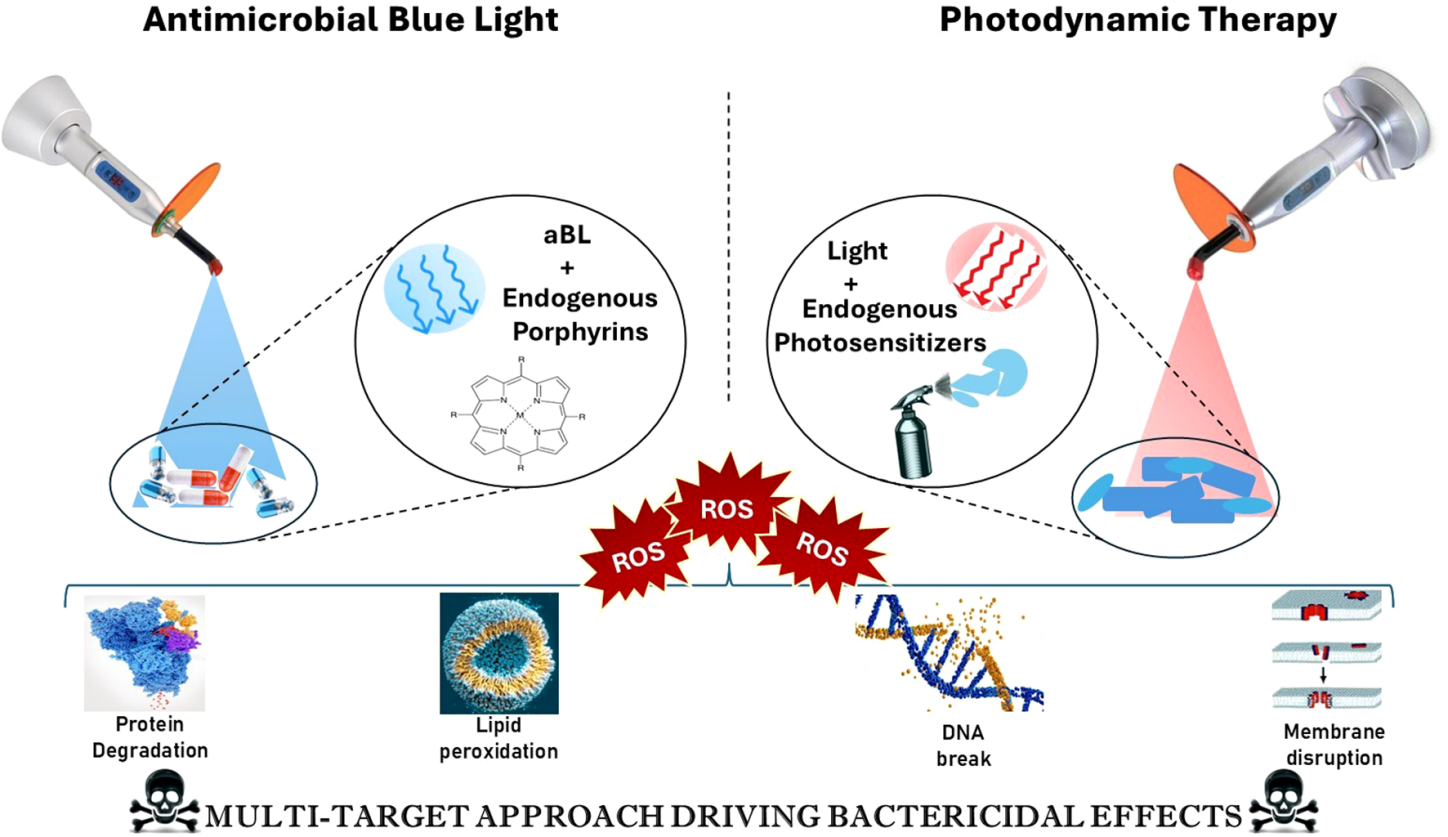
Mechanism of action of antimicrobial blue light (aBL) and photodynamic therapy (PDT) in bacterial inactivation. [Antimicrobial Blue Light (left) activates endogenous porphyrins in microbial cells, generating reactive oxygen species (ROS) that contribute to protein degradation and lipid peroxidation. Photodynamic Therapy (right) involves the use of exogenous photosensitizers activated by red light, leading to ROS generation that causes DNA damage and membrane disruption. Both approaches converge on a multi-target bactericidal mechanism through oxidative stress, ultimately enhancing microbial cell death].

The alarming rise of MDR bacteria has significantly strained current antimicrobial therapies, necessitating the exploration of innovative strategies that challenge traditional resistance mechanisms. The antimicrobial potential of blue light has been recognized for over two decades. Early research showed that visible blue light at a wavelength of 405 nm could effectively kill methicillin-resistant Staphylococcus aureus (MRSA) *in vitro* (Enwemeka et al., 2008). This finding laid the foundation for the development of Antimicrobial Blue Light (aBL) therapies, which utilize the photoactivation of endogenous porphyrins within bacterial cells to produce reactive oxygen species (ROS). These ROS cause oxidative damage to essential biomolecules, including membranes, proteins, and DNA, ultimately leading to bacterial death. As shown in **Figure 1**, the mechanism involves the ROS-mediated breakdown of proteins, lipid peroxidation, DNA fragmentation, and membrane disruption. This broad-spectrum, multi-targeted approach reduces the risk of resistance development, making aBL a promising tool against multidrug-resistant (MDR) pathogens. Importantly, this mode of action is broad-spectrum and resilient against the development of resistance due to its multi-faceted approach. PDT represents another promising approach in this field, utilizing similar principles but employing exogenous photosensitizers that generate highly ROS when activated by a specific wavelength of light. These photosensitizers tend to accumulate in bacterial cells preferentially, allowing for selective destruction upon exposure to light (Cieplik et al., 2014; Leanse et al., 2023; Teng et al., 2023).

In this context, the terms “endogenous photosensitizers” and “endogenous porphyrins” are not entirely interchangeable. Endogenous photosensitizers include porphyrins as well as other chromophores naturally found in microbial cells, such as flavins and NADH. These molecules absorb blue light and generate reactive oxygen species (ROS) during antimicrobial blue light (aBL) therapy. On the other hand, exogenous photosensitizers—like methylene blue, SAPYR, and chlorin e6—are administered externally and activated by specific light wavelengths in photodynamic therapy (PDT). The light used in PDT can include blue, red, or near-infrared wavelengths. Both PDT and aBL therapies lead to oxidative damage in cellular membranes, lipids, proteins, and DNA. As a result, processes such as lipid peroxidation, protein degradation, and DNA fragmentation are not exclusive to one treatment modality; rather, they are shared consequences of ROS generation that depend on the type of sensitizer used and the amount of light applied (Cieplik et al., 2014; Przygoda et al., 2023).

**Figure 2:**
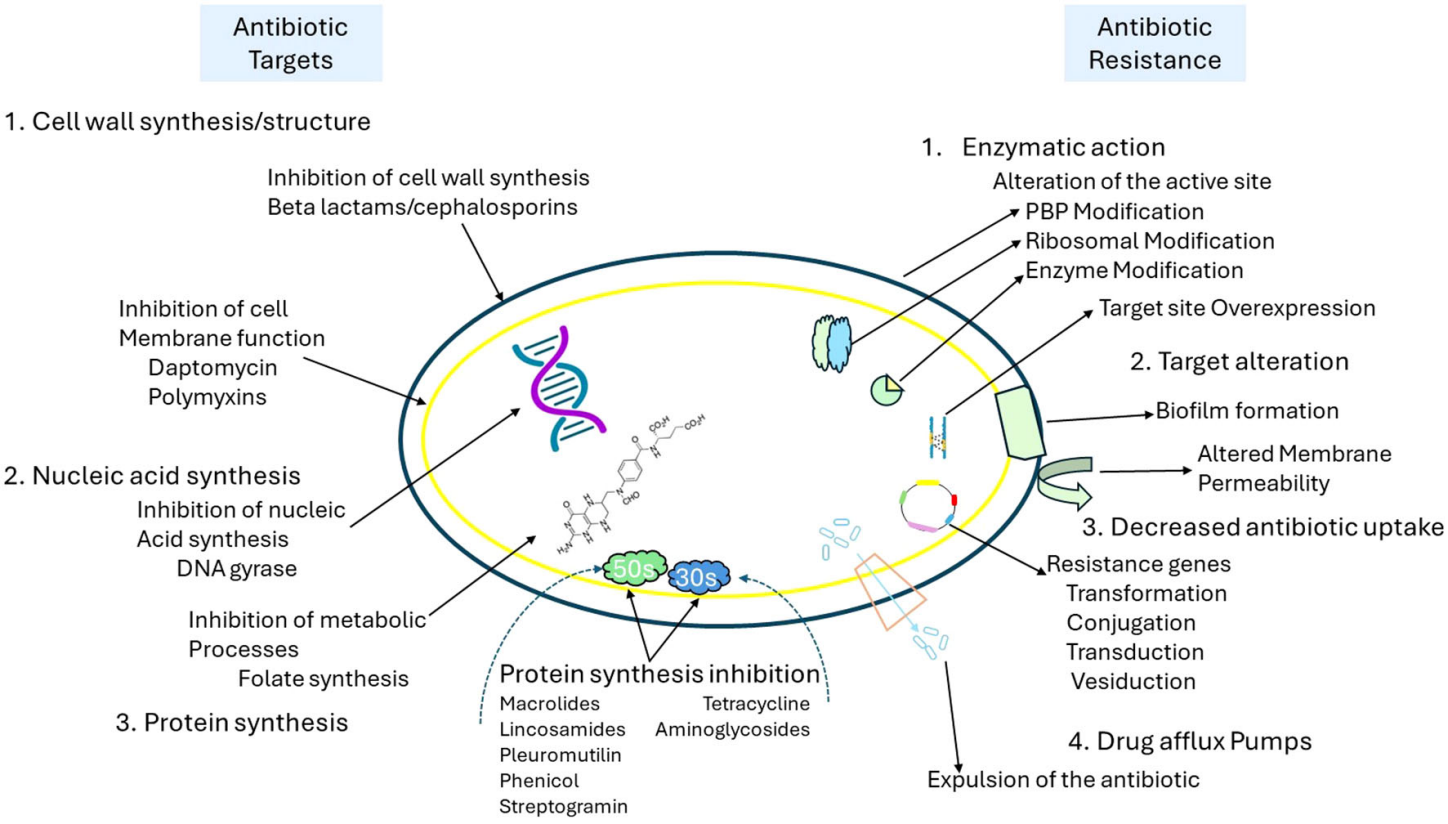
Overview of antibiotic targets and mechanisms of antibiotic resistance in bacteria. [This schematic illustrates key bacterial targets for antibiotics (left) and corresponding resistance mechanisms (right). Antibiotics act by inhibiting cell wall synthesis (e.g., beta-lactams), disrupting membrane function (e.g., daptomycin, polymyxins), inhibiting nucleic acid synthesis (e.g., DNA gyrase inhibitors), and blocking protein synthesis (e.g., macrolides, tetracyclines). In contrast, bacteria employ multiple resistance strategies, including enzymatic modification of antibiotic targets (e.g., PBP and ribosomal alterations), target site overexpression, biofilm formation, altered membrane permeability, reduced antibiotic uptake via genetic transfer mechanisms (e.g., transformation, conjugation), and drug efflux through specialized pumps that expel antibiotics from the cell].

Traditional antibiotic approaches, including β-lactams, macrolides, and fluoroquinolones, usually target critical microbial processes such as cell wall synthesis, protein translation, and DNA replication. However, the effectiveness of these antibiotics is increasingly compromised by bacterial resistance mechanisms, which include enzymatic degradation, efflux pumps, and modifications to the target sites, as shown in **Figure 2**. In response to the growing issue of antimicrobial resistance, new strategies have been developed to bypass or overcome these resistance mechanisms. These strategies include bacteriophage therapy, antimicrobial peptides (AMPs), anti-virulence agents that block quorum sensing, CRISPR-based antimicrobials, and light-based therapies such as photodynamic therapy (PDT) and antimicrobial blue light (aBL). Unlike traditional antibiotics, these innovative approaches operate through unique mechanisms, including physical disruption of membranes, generation of reactive oxygen species (ROS), and genetic interference, which decreases the likelihood of developing resistance. Traditional antibiotic strategies are increasingly challenged by bacterial resistance mechanisms as illustrated in **Figure 2**, including enzymatic degradation, alterations at target sites, efflux pumps, and reduced permeability. PDT circumvents these resistance mechanisms by attacking bacterial components through phototoxicity, demonstrating effectiveness even against multidrug-resistant (MDR) strains. By steering clear of conventional antibiotic targets such as ribosomes, DNA gyrase, and enzymes associated with the cell wall, PDT minimizes selective pressure and delays the emergence of resistance, making it an appealing alternative for the post-antibiotic era (Cieplik et al., 2013; Murray et al., 2022; Zhao et al., 2024).

## 2 Photodynamic therapy

### 2.1 Key components of PDT

#### 2.1.1 Photosensitizers

Photosensitizers are molecules that become activated by light and produce reactive oxygen species (ROS) upon excitation. Ideal characteristics of these molecules include high singlet oxygen quantum yields, low toxicity in the absence of light, and an absorption profile that aligns well with therapeutic light sources. While wavelengths between 600 nm and 800 nm are often highlighted for their ability to penetrate deep tissues, they are not the best choice for all applications in photodynamic therapy (PDT). For example, recent studies have shown that photosensitizers like SAPYR can be effectively activated by blue light (approximately 460 nm) and act as exclusive Type-II singlet oxygen generators. This is particularly important in antimicrobial photodynamic therapy (aPDT), where deep tissue penetration is not as critical (Cieplik et al., 2013, 2015). It is important to distinguish between the therapeutic targets and requirements of tumor photodynamic therapy (PDT) and antimicrobial photodynamic therapy (aPDT). In tumor therapy, photosensitizers typically accumulate in cancerous tissue through the enhanced permeability and retention (EPR) effect or through targeted delivery. These photosensitizers remain inactive until activated by localized light exposure. In contrast, aPDT relies on rapid physicochemical interactions, such as cationic photosensitizers binding electrostatically to the negatively charged cell walls of bacteria, and does not require long-term retention. As a result, the design, activation wavelength, and pharmacokinetics of photosensitizers should be specifically tailored to their intended application. aPDT may benefit from shorter wavelengths (e.g., blue light) and surface-level targeting, while oncologic PDT focuses on achieving deeper tissue penetration and tumor selectivity (Cieplik et al., 2013).

#### 2.1.2 Light activation

Effective photodynamic therapy (PDT) relies on light of a specific wavelength that matches the absorption peak of the chosen photosensitizer. Common light sources include lasers and light-emitting diodes (LEDs), which allow for adjustments in intensity and spectral output. Proper dosage is critical; insufficient light may not activate the photosensitizer effectively, while excessive exposure could harm surrounding tissues. The fluence rate and total light dose directly influence the generation of reactive oxygen species (ROS) and, therefore, the therapy’s effectiveness. Notably, the depth of light penetration into tissues is dependent on its wavelength, with near-infrared light (650–800 nm) penetrating more deeply. A vital factor to consider is the number of absorbed photons, which fundamentally affects ROS production and the overall biological outcome (Algorri et al., 2023; Cieplik et al., 2015).

#### 2.1.3 Oxygen-dependent mechanisms

The efficiency of PDT depends on the presence of molecular oxygen in tissues. A photosensitizer transforms oxygen molecules into very reactive singlet oxygen by transferring energy to them when it is triggered by light. Cell death in target tissues is caused by this reactive species. Low oxygen levels, or hypoxia, can reduce PDT efficiency, therefore critical to maintain proper oxygenation throughout treatment (Cieplik et al., 2013).

**Figure 3:**
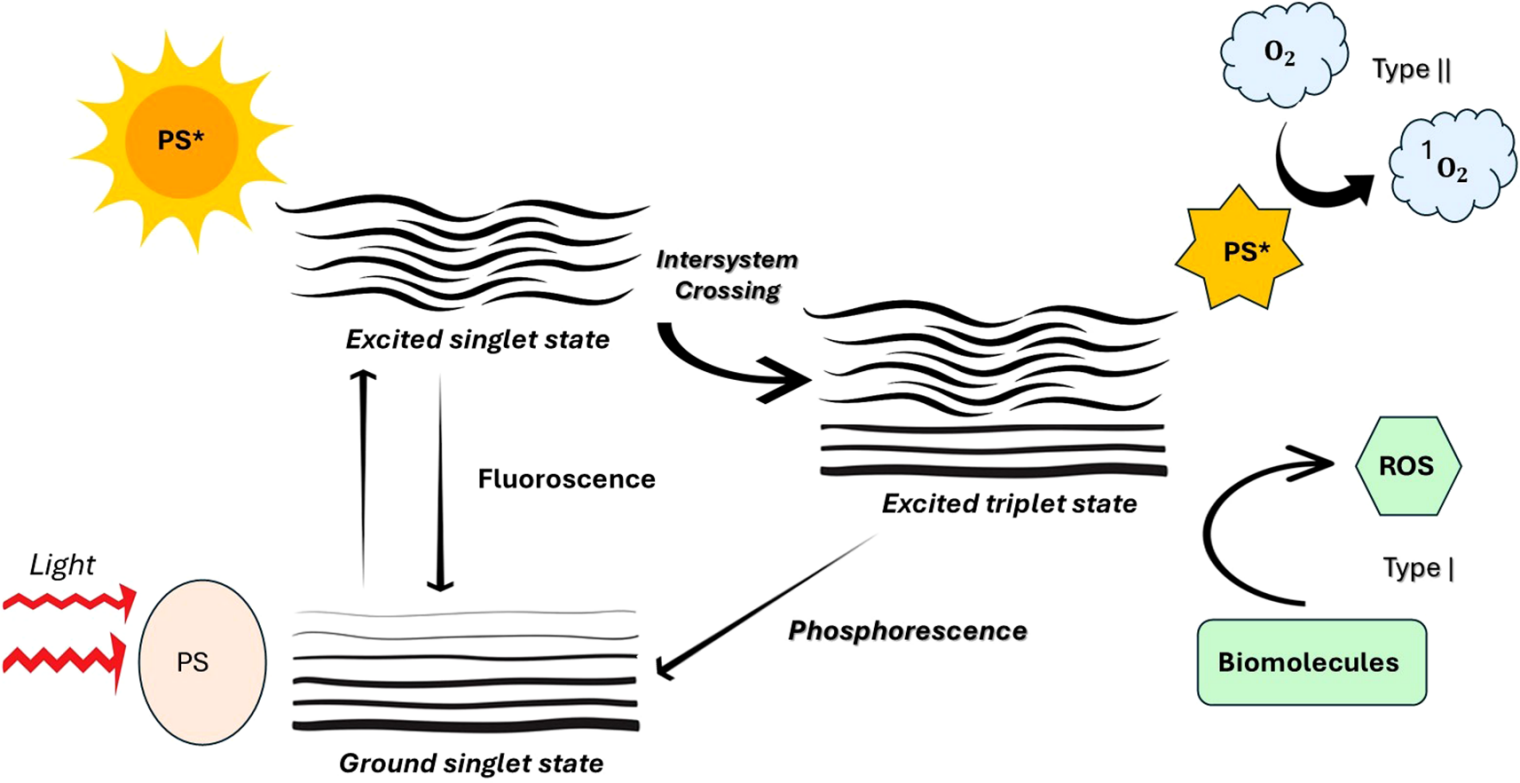
Photophysical and photochemical mechanisms of photosensitizer activation in photodynamic therapy (PDT). [Upon absorption of light, the photosensitizer (PS) transitions from the ground singlet state to an excited singlet state. It can return to the ground state by emitting fluorescence or undergo intersystem crossing to form an excited triplet state. From this triplet state, the PS can generate reactive oxygen species (ROS) through two pathways: Type I, involving electron transfer reactions that produce ROS (e.g., superoxide or hydroxyl radicals) that interact with biomolecules, and Type II, involving energy transfer to molecular oxygen (O_2_) to produce singlet oxygen (¹O_2_). Additionally, the excited triplet state may return to the ground state via phosphorescence. These ROS mediate the cytotoxic effects central to PDT].

The **Figure 3** depicts the photodynamic process occurs through two main mechanisms: Type I and Type II. During excitation, the photosensitizer may either transfer electrons or hydrogen (Type I), resulting in the production of superoxide anions (O_2_•−), hydrogen peroxide (H_2_O_2_), and hydroxyl radicals (•OH). While Type I is more effective under low-oxygen (hypoxic) conditions, it still necessitates the presence of molecular oxygen. Conversely, in the Type II pathway, energy is transferred directly to molecular oxygen, leading to the formation of singlet oxygen (¹O_2_), which is a highly reactive and cytotoxic species. Both pathways contribute to oxidative stress and cell damage in targeted microbial or cancerous tissues (Harris and Rajasekar, 2025; Dube, 2024; Warszyńska et al., 2023).

**Table 1**. illustrates the effectiveness of different photosensitizers utilized in photodynamic therapy (PDT) targeting a variety of multidrug-resistant (MDR) pathogens. The chosen pathogens consist of both Gram-positive and Gram-negative bacteria, in addition to resistant fungal strains. Methylene Blue stands out due to its wide-ranging effectiveness and successful treatment against several resistant organisms, such as Staphylococcus aureus (MRSA), Klebsiella pneumoniae, and Candida albicans. Other photosensitizers, including Rose Bengal, Curcumin, and Hypericin, also showed significant results against pathogens like vancomycin-resistant Enterococcus faecalis and penicillin-resistant Streptococcus pneumoniae. The results of PDT showed a range of microbial inactivation from moderate to high, influenced by the type of organism, the photosensitizer used, and its resistance characteristics. This table highlights the flexibility of PDT in addressing conventional resistance mechanisms and reinforces its potential as a promising supplemental or alternative approach for tackling MDR infections (Aroso et al., 2021; Ghorbani et al., 2018).

**Table 1:** Selected photosensitizers and their characteristics for PDT applications.

No.	Photosensitizer	Chemical class	Charge	Absorption maximum	Bacterial targets	Reference
1	Methylene Blue	Synthetic Dye	Cationic	632 nm	Dental plaque, E. coli	(Ghorbani et al., 2018)
2	Toluidine Blue	Synthetic Dye	Cationic	410 nm	S. mutans, E. coli	(Ghorbani et al., 2018; Wainwright and Crossley, 2004)
3	Rose Bengal	Synthetic Dye	Anionic	532 nm	E. faecalis, P. aeruginosa	(Ghorbani et al., 2018; Nakonieczna et al., 2018)
4	Curcumin	Natural Compound	Neutral	547 nm	S. mutans, L. acidophilus	(Ghorbani et al., 2018; Wainwright and Crossley, 2004)
5	Hypericin	Natural Compound	Neutral	593 nm	S. aureus, E. coli	(Ghorbani et al., 2018; Maisch, 2007)
6	TMPyP (Porphyrin derivative)	Tetra-pyrrole	Cationic	446 nm	S. aureus, P. aeruginosa	(Ghorbani et al., 2018)
7	Zinc Phthalocyanine	Tetra-pyrrole	Neutral	670 nm	A. hydrophila, S. aureus	(Ghorbani et al., 2018; Wainwright and Crossley, 2004)
8	Chlorin e6	Tetra-pyrrole	Neutral	660 nm	S. aureus, E. coli	(Ghorbani et al., 2018; Maisch, 2007)
9	Fullerenes	Nanostructure	Neutral	532 nm	S. aureus, E. coli	(Ghorbani et al., 2018; Dai et al., 2009)
10	Titanium Dioxide	Nanostructure	Neutral	Near-UV (400 nm)	Water treatment	(Ghorbani et al., 2018; Dai et al., 2009)

### 2.2 Interaction of PDT with pathogens

#### 2.2.1 Effects of PDT on bacterial and fungal cells

A novel antibacterial treatment called PDT uses the interplay of photosensitizers and light to create ROS. In both bacteria and fungus, these ROS cause oxidative stress, which harms cellular constituents like membranes and DNA. PDT successfully breaks down biofilms produced by bacterial infections caused by species such as Pseudomonas aeruginosa and Staphylococcus aureus, which are infamously resistant to traditional therapies (Suresh et al., 2024). By exposing the germs to the host’s immune system, this disruption not only directly kills the bacteria but also makes them more vulnerable to subsequent treatments (Polat and Kang, 2021).

As seen with Candida species, PDT also affects fungal cells by weakening their cell walls and affecting their metabolic functions (Lima et al., 2019). In order to effectively combat bacterial infections, PDT also recruits neutrophils, which alters the immunological response (Malech et al., 2014). PDT’s dual mechanism of immune system stimulation and direct microbial death makes it an effective technique for fighting resistant microbial illnesses (Zhang et al., 2025).

#### 2.2.2 Disruption of cellular membranes and genetic material

##### 2.2.2.1 Cellular membrane distribution

PDT causes considerable harm to cellular membranes due to the oxidative stress produced by reactive oxygen species (ROS). Photosensitizers like polycation-containing hematoporphyrin tend to accumulate at negatively charged membrane glycans, resulting in lipid peroxidation and the breakdown of structural integrity (Przygoda et al., 2023).

**Table 2**. presents a comparative study of frequently researched photosensitizers utilized in antimicrobial PDT, detailing their chemical classifications, charge characteristics, absorption peaks, and targeted microorganisms. This compilation features synthetic dyes such as Methylene Blue and Toluidine Blue, in addition to natural substances like Curcumin and Hypericin. Importantly, the most effective photosensitizers tend to possess cationic or neutral charges, which enhances their affinity for negatively charged microbial membranes. The absorption maxima, which span from the visible to near-UV spectra, play a crucial role in ensuring optimal light activation and the generation of reactive oxygen species (ROS). Tetra-pyrrole compounds, including Chlorin e6 and Zinc Phthalocyanine, demonstrate strong absorbance within the red/near-infrared range, facilitating deeper tissue penetration. The properties of photosensitizers affect their effectiveness against different pathogens, such as E. coli, S. aureus, and P. aeruginosa. This table demonstrates how the choice of photosensitizers, guided by their physicochemical characteristics, can be customized according to the type of microbe and the site of infection, highlighting the adaptability of a PDT in clinical settings (González et al., 2021; da Fonseca et al., 2021; Dai et al., 2009; García et al., 2015; Ghorbani et al., 2018; Rubilar-Huenchuman et al., 2024; Wainwright and Crossley, 2004).

**Table 2:** MDR pathogen sensitivity to PDT across diverse photosensitizers.

No.	MDR pathogen	Strain type	Resistance profile	Photosensitizer used	PDT outcome	Known resistance mechanism(s)	Reference
1	*Staphylococcus aureus* (MRSA)	Gram-positive	Methicillin-resistant	Methylene Blue	Significant reduction	Altered PBPs, Efflux pumps	(Dai et al., 2009; Reygaert, 2009)
2	*Pseudomonas aeruginosa*	Gram-negative	Carbapenem-resistant	Toluidine Blue O	Partial reduction	Efflux pumps, Porin loss, Enzymatic degradation	(Wainwright and Crossley, 2004)
3	*Acinetobacter baumannii*	Gram-negative	Carbapenem-resistant	Methylene Blue	Significant reduction	Efflux pumps, Enzymatic inactivation (OXA-type β-lactamase)	(da Fonseca et al., 2021)
4	*Enterococcus faecalis* (VRE)	Gram-positive	Vancomycin-resistant	Rose Bengal	Effective under low light dose	Altered target (D-Ala-D-Lac), Efflux pumps	(Rubilar-Huenchuman et al., 2024)
5	*Escherichia coli* (ESBL)	Gram-negative	Extended-spectrum β-lactamase producer	Curcumin	Moderate reduction	ESBLs (enzymatic inactivation), Efflux pumps	(Ghorbani et al., 2018)
6	*Klebsiella pneumoniae*	Gram-negative	Carbapenemase-producing (KPC, ESBL)	Methylene Blue	High inactivation rate	Enzymatic inactivation, Efflux pumps, Porin mutations	(Buchovec et al., 2022)
7	*Streptococcus pneumoniae*	Gram-positive	Penicillin-resistant	Hypericin	Effective inhibition	Altered PBPs, Efflux pumps (mefA)	(García et al., 2015)
8	*Enterococcus faecium*	Gram-positive	Vancomycin-resistant	Methylene Blue	Significant reduction	Target modification (vanA/vanB), Efflux pumps	(Dai et al., 2009)
9	*Klebsiella aerogenes*	Gram-negative	Carbapenem-resistant	Methylene Blue	Effective inhibition	Enzymatic inactivation (AmpC β-lactamase), Efflux pumps	(Buchovec et al., 2022)
10	*Candida albicans*	Fungal	Fluconazole-resistant	Methylene Blue	Significant reduction	Efflux pumps (CDR1, MDR1), Ergosterol target alteration	(Dai et al., 2009; de Carvalho Leonel et al., 2019)
11	*Cryptococcus neoformans*	Fungal	Amphotericin B-resistant	Methylene Blue	Effective inhibition	Membrane alterations, Efflux pumps (ABC transporters)	(Dai et al., 2009)
12	*Staphylococcus epidermidis*	Gram-positive	Methicillin-resistant	Methylene Blue	Significant reduction	Altered PBPs, Efflux pumps	(García et al., 2015)
13	*Enterobacter cloacae*	Gram-negative	Carbapenem-resistant	Methylene Blue	Effective inhibition	Enzymatic inactivation, Porin loss, Efflux pumps	(Buchovec et al., 2022)
14	*Proteus mirabilis*	Gram-negative	Ampicillin-resistant	Methylene Blue	Significant reduction	β-lactamase production, Efflux pumps	(García et al., 2015; Reygaert, 2018)

### 2.3 PDT in microbial treatment

The distinctions between the impact of PDT on microbial resistance and that of conventional therapies are essential and arise from their differing modes of action. Conventional therapies, including antibiotics and antifungals, generally focus on specific cellular functions or structures within microorganisms, such as the synthesis of cell walls, replication of DNA, or production of proteins. Microorganisms are capable of developing resistance to these treatments through various strategies, such as enzymatically deactivating the drug, modifying the drug’s target, using efflux pumps to expel the drug from the cell, and decreasing cell permeability. These resistance mechanisms typically necessitate only a handful of genetic modifications, allowing for a relatively rapid emergence of resistance (Guo et al., 2018).

In contrast, PDT employs a non-specific oxidative approach. When energized by light, the photosensitizer produces highly ROS—like singlet oxygen—that inflict extensive damage to various cellular components, such as lipids, proteins, and nucleic acids. This broad, non-targeted assault makes it significantly more challenging for microorganisms to acquire resistance. Developing resistance would necessitate simultaneous and coordinated alterations in multiple cellular pathways to defend against the oxidative harm induced by ROS. Although some microorganisms may show a degree of tolerance by boosting antioxidant production or enhancing DNA repair mechanisms, the emergence of strong resistance to PDT is deemed much less probable compared to conventional therapies due to the intricate and multifaceted nature of PDT’s cytotoxic actions. This distinction is a primary reason why PDT is being investigated as a viable option to address the escalating issue of multidrug resistance (Liu et al., 2015; Surur, et al., 2024).

## 3 Mechanisms of multidrug resistance and how PDT overcomes them

### 3.1 MDR in microbial infections

MDR among microbes poses a significant threat to global health. Microorganisms acquire the ability to withstand multiple antimicrobial treatments through different mechanisms, complicating the treatment of infections. Gaining insight into these resistance mechanisms is essential for developing new strategies to address MDR and maintain the efficacy of current medications (Salam et al., 2023).

The main mechanisms of multidrug resistance (MDR) include efflux pumps, enzymatic degradation, modification of target sites, reduced permeability, and biofilm formation. These mechanisms can vary across different microbial strains, and not all pathogens listed in **Table 2** necessarily utilize efflux pumps. The excessive use of antibiotics promotes the dissemination of these resistance mechanisms, highlighting the urgent need for innovative therapeutic solutions (Ahmed et al., 2024; Elshobary et al., 2025).

### 3.2 PDT as a potential solution

PDT overcomes prevalent MDR strategies through its distinct mode of action. Unlike traditional antimicrobial or chemotherapy agents that focus on particular cellular targets, PDT operates by producing ROS, resulting in extensive oxidative harm to various cellular elements. This non-specific approach effectively evades resistance mechanisms like enzymatic breakdown and modified target sites, as the ROS directly harm critical cellular components irrespective of any resistance factors present. Furthermore, PDT can counteract the impact of efflux pumps, a prevalent mechanism of MDR, by producing ROS within the cells. The production of these intracellular ROS damages crucial cellular components, regardless of the concentration of the medication. In addition, the oxidative stress induced by PDT can harm or deactivate the proteins of efflux pumps, which further boosts its effectiveness against MDR strains. This multifaceted strategy positions PDT as a viable alternative to conventional therapies in the fight against MDR (Alwaeli et al., 2015; Alfei et al., 2024; Liu et al., 2015).

**Figure 4:**
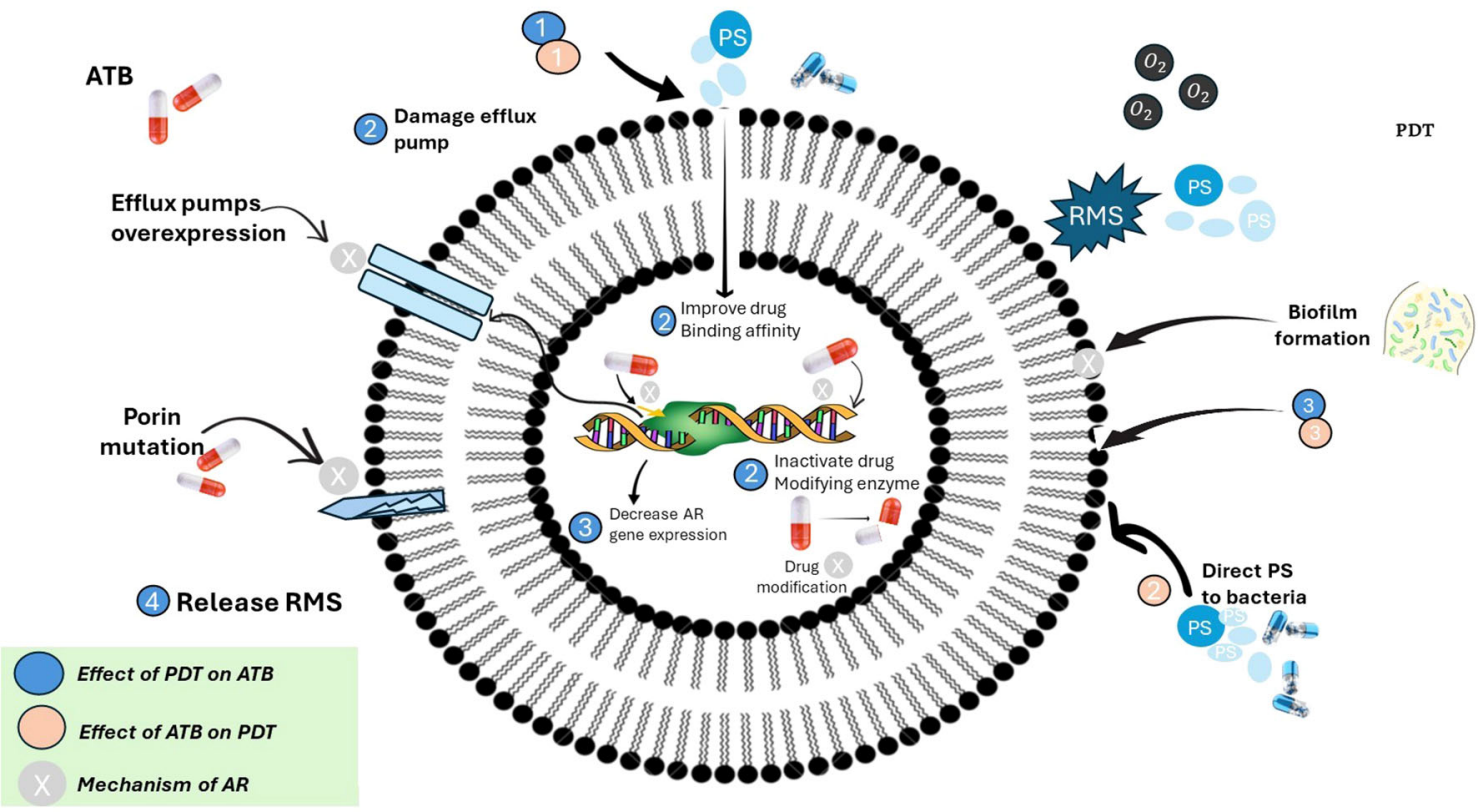
Interplay between photodynamic therapy (PDT), antibiotics (ATB), and antimicrobial resistance (AR) mechanisms in bacteria. [This schematic illustrates the complex interactions between antibiotics (ATB), photodynamic therapy (PDT), and bacterial antimicrobial resistance (AR). Antibiotic resistance mechanisms (indicated by beige circles) include overexpression of efflux pumps, porin mutations, drug-modifying enzymes, and biofilm formation, all of which reduce antibiotic efficacy. PDT (blue circles) exerts its effect through reactive molecular species (RMS), particularly reactive oxygen species (ROS), which can: (1) damage efflux pumps, (2) inactivate drug-modifying enzymes, (3) disrupt biofilms, (4) release RMS to reduce AR gene expression and improve antibiotic binding affinity. Conversely, antibiotics can enhance PDT by directing photosensitizers (PS) to bacterial targets. The figure emphasizes a synergistic strategy where PDT can overcome antibiotic resistance, and antibiotics can potentiate PDT effectiveness. Color-coded legend: blue = effect of PDT on antibiotics; beige = effect of antibiotics on PDT; grey = intrinsic mechanisms of antibiotic resistance].

**Figure 4** depicts the established ways in which bacteria develop resistance to traditional antibiotics, including the use of efflux pumps, enzymatic drug inactivation, and modifications to antibiotic target sites. In contrast, the figure also demonstrates that photodynamic therapy (PDT) combats microbes by generating reactive oxygen species (ROS), which inflict widespread oxidative damage on bacterial membranes, proteins, and genetic material. Notably, there are no known cases of stable or heritable resistance to PDT. While some bacteria may temporarily adapt by increasing their tolerance to oxidative stress, this response does not equate to the genetically encoded resistance seen with antibiotics. Because PDT’s oxidative damage targets multiple cellular components and bypasses conventional resistance pathways, it stands out as a promising approach for managing multidrug-resistant infections (Feng et al., 2022, 2022; Tavares et al., 2010).

Exploring non-traditional pathways to target microbial cells presents a promising opportunity to address antimicrobial resistance and create innovative therapeutic strategies. Conventional antimicrobial agents typically aim at specific bacterial functions, such as the synthesis of cell walls, DNA replication, or protein synthesis. Nevertheless, microorganisms are capable of developing resistance mechanisms that can make these agents ineffective. Non-traditional methods emphasize disrupting microbial survival through alternative means, like targeting virulence factors, interfering with quorum sensing, or inhibiting metabolic pathways critical for their survival. Another strategy involves photodynamic therapy, where the production of reactive oxygen species causes damage to various cellular components. These methods bypass existing resistance mechanisms and suggest a potential solution to the escalating challenge of AMR (Murugaiyan et al., 2022; Feng et al., 2022).

## 4 Photodynamic therapy against multidrug-resistant microorganisms

### 4.1 PDT for resistant bacterial infections

PDT has proven effective against a wide range of bacteria, which includes both Gram-positive and Gram-negative strains, as well as those resistant to antibiotics. While there are significant differences in the cell wall composition between Gram-positive and Gram-negative bacteria—specifically, the outer membrane of Gram-negative bacteria can hinder the absorption of certain substances—PDT has shown potential against both groups. The ROS produced during PDT can inflict damage on various cellular components regardless of cell wall structure, resulting in bacterial inactivation. This extensive spectrum of activity makes PDT a significant asset in addressing infections caused by various pathogens (Sperandio et al., 2013).

The ability of PDT to combat antibiotic-resistant bacteria is especially remarkable. Mechanisms of antibiotic resistance, including efflux pumps, enzymatic breakdown, and modifications of target sites, often make conventional antibiotics ineffective. Nevertheless, PDT can bypass these issues by producing ROS that induce widespread oxidative damage to a range of cellular components. Furthermore, PDT can penetrate resistant cells found within biofilms, effectively addressing the protective barrier that contributes to antibiotic resistance. Research has indicated that PDT can successfully eliminate antibiotic-resistant bacterial strains such as methicillin-resistant Staphylococcus aureus (MRSA), vancomycin-resistant Enterococci (VRE), and multidrug-resistant Pseudomonas aeruginosa, underscoring its potential as an innovative treatment approach to tackle the rising challenge of antibiotic resistance (Liu et al., 2015).

### 4.2 PDT in fungal infection

PDT has demonstrated encouraging outcomes in the fight against Candida species and other resistant fungi, presenting a possible alternative to standard antifungal treatments. Fungal infections, particularly those caused by Candida species, pose a significant threat, especially for individuals with weakened immune systems. The rise of antifungal-resistant strains makes treatment approaches even more challenging. The action mechanism of PDT, which includes the production of ROS, can effectively target fungal cells regardless of their resistance traits. ROS can harm fungal cell membranes, cell walls, and internal components, resulting in cell death. PDT has proven effective against various Candida species, including Candida albicans, along with other resistant fungi such as Aspergillus species. Therefore, PDT stands out as a useful method for treating fungal infections that do not respond to traditional antifungal medications (Hung et al., 2021; Rodríguez-Cerdeira et al., 2021a; X. Wu and Hu, 2022).

## 5 Synergy between PDT and antibiotics

Combination therapies that include PDT present a promising approach for increasing effectiveness against resistant bacterial and fungal strains. By pairing PDT with other antimicrobial agents, such as traditional antibiotics or antifungals, synergistic effects can be achieved, leading to better elimination of resistant microorganisms. The logic behind these combination therapies is that PDT can undermine the resistance mechanisms of microorganisms, making them more vulnerable to the actions of additional antimicrobial agents. For instance, PDT can break down biofilms, which can help antibiotics penetrate the biofilm matrix more effectively. Moreover, PDT can impair efflux pumps, resulting in higher intracellular levels of antibiotics. Additionally, the integration of PDT with immunomodulatory agents can enhance the host’s immune response, further improving the elimination of resistant microorganisms (Akhtar et al., 2024; Pérez-Laguna et al., 2021b).

## 6 Clinical evidence and case studies

Photodynamic therapy (PDT) has shown effectiveness in treating various resistant infections in both clinical and preclinical studies. For instance, PDT using methylene blue has been successfully applied to wounds infected with methicillin-resistant Staphylococcus aureus (MRSA). This treatment resulted in faster healing and a reduction of over 80% in bacterial load (Dai et al., 2009). PDT has proven effective in treating Pseudomonas aeruginosa infections in burn wounds and ulcers, as well as addressing fungal infections like oral and vaginal candidiasis caused by Candida albicans (MaChado-de-Sena et al., 2014; Rodríguez-Cerdeira et al., 2021b). Additionally, PDT has been utilized in the treatment of fungal keratitis, onychomycosis, and localized cutaneous leishmaniasis, yielding encouraging results and few adverse effects (Ramos et al., 2016; Hung et al., 2021).

### 6.1 Pre-clinical and clinical studies in PDT

#### 6.1.1 Preclinical innovations in PDT

Next-era photosensitizers and targeting techniques are revolutionizing PDT through enhancing precision and efficacy against multidrug-resistant pathogens. Indium phosphide (InP) quantum dots generate bactericidal superoxide radicals, eradicating MDR Staphylococcus aureus in murine wounds without harming mammalian cells, at the same time as hybrid nano systems like silver nanoparticle-rhodamine B complexes leverage photothermal-photodynamic synergy to get rid of 98% of E. coli and MRSA inside 5 mins of light exposure. Stimuli-responsive companies, which includes pH-activated porphyrin-metallic natural frameworks, selectively release ROS in acidic contamination microenvironments, sparing healthful tissues. targeting techniques further refine PDT’s specificity: cationic polymers like Amberlite resin-changed Rose Bengal triumph over hydrophobicity to improve Gram-negative bacterial focused on, and antibody-conjugated photosensitizers, including anti-Pseudomonas monoclonal antibodies guiding chlorin e6, reduce lung infection bacterial loads by means of four-log in preclinical fashions. Combinatorial strategies extend these outcomes—gold nanorods permit concurrent photothermal therapy and PDT to eliminate Acinetobacter baumannii biofilms at reduced light doses, even as PDT-mediated disruption of efflux pumps in Klebsiella pneumoniae restores susceptibility to colistin *in vitro*, resensitizing pathogens to traditional antibiotics. These innovations together decorate PDT’s potential as a focused, multifunctional weapon towards resistant infections (Lee et al., 2022; G. Wu et al., 2024).

#### 6.1.2 Clinical innovations in PDT

Advances in photodynamic therapy (PDT) emphasize new photosensitizers and enhanced delivery methods, while targeting real-world multidrug-resistant infections. In instances of onychomycosis, MAL-PDT has completed therapy fees of as much as 90%, outperforming traditional antifungals in sufferers mistaken for systemic remedies. Methylene blue-based photodynamic therapy (PDT) has demonstrated significant benefits in clinical studies for patients with persistent ulcers infected with Staphylococcus aureus. This therapy accelerates wound closure, reduces bacterial counts, and enhances tissue repair. For instance (Dai et al., 2009),patients who received PDT achieved more than 80% bacterial clearance and experienced faster epithelial healing within 10 days. In contrast, those treated with mupirocin showed a slower recovery. Important clinical advancements include the use of device coatings and the integration of photodynamic therapy into textiles. PDT has additionally proven efficacy towards viral pathogens like cutaneous HPV and HSV by way of interfering with viral replication cycles, and in parasitic illnesses which includes localized leishmaniasis, wherein topical PDT has finished 100% treatment quotes without the systemic side outcomes related to conventional medicinal drugs. collectively, these advances underscore the extensive-spectrum and flexible applications of PDT in combating multidrug-resistant and hard-to-treat infections (Ramos et al., 2016; G. Wu et al., 2024).

## 7 Challenges and limitations of photodynamic therapy

Even with its potential benefits, the broad implementation of PDT encounters numerous obstacles. A major challenge is light penetration, as the light must reach the photosensitizer for activation, which is especially difficult for infections or tumors located deeper within the body. Approaches to address this challenge include utilizing higher light doses (with safety considerations), employing photosensitizers that respond to longer wavelengths of light (which can reach deeper tissues), or applying alternative light delivery techniques such as interstitial illumination (Wainwright and Crossley, 2004). The properties of photosensitizers also present challenges; optimal photosensitizers should have high selectivity for targeted cells, low toxicity to surrounding healthy tissues, and effective activation from easily accessible light sources. Moreover, although the risk of developing resistance to PDT is generally perceived to be lower than that associated with conventional antimicrobials due to its multi-targeting nature, it remains a potential issue that should be monitored and examined. Lastly, the financial aspect and availability of PDT are vital factors influencing its general acceptance. PDT equipment and photosensitizers can be costly, and specialized knowledge is necessary to administer the treatment. Enhancing the cost-effectiveness and expanding the availability of PDT could make it a more feasible treatment choice for patients globally (Ahmed et al., 2024; Shi et al., 2019).

## 8 Future directions and innovations

The prospects for PDT in treating MDR infections are encouraging, with several innovative pathways emerging. Progress in the development of photosensitizers, especially through advancements in nanotechnology and new formulations, is improving the targeting of microbial cells while minimizing side effects on healthy tissues. This enhanced selectivity broadens the therapeutic window of PDT. Additionally, there is an exploration of PDT’s potential in personalized medicine, where treatment protocols and photosensitizers are customized to align with the infection’s specific characteristics and the patient’s needs, maximizing effectiveness and reducing off-target impacts (Hamblin and Hasan, 2004; Liu et al., 2015). The combination of PDT with other therapeutic approaches, such as traditional antibiotics or immunomodulatory agents, shows great potential for boosting treatment efficacy against resistant strains. Current trends in both clinical and preclinical research involve the examination of new photosensitizers, improved light delivery techniques, and novel treatment protocols for various MDR infections, setting the stage for PDT to play a significant role in combating antibiotic resistance (Harris and Rajasekar, 2025; Miranda et al., 2018).

## 9 Conclusion

In conclusion, PDT offers a promising and novel solution to address the increasing issue of MDR in microbial infections. Its distinct mode of action, which involves the production of reactive oxygen species to cause extensive cellular damage, enables it to bypass many of the resistance mechanisms that make traditional antibiotics and antifungals ineffective. As research and development progress in optimizing PDT methods and photosensitizers, its application in existing clinical settings becomes more practical. PDT can be used alongside conventional treatments, particularly for localized MDR infections or as a strategy to eliminate resistant strains. In the future, PDT shows great potential as a sustainable and adaptable option in the battle against MDR, providing hope in a scenario where antimicrobial resistance continues to rise. Ongoing research, clinical studies, and technological innovations are essential to fully realize the capabilities of PDT and to establish it as a fundamental element of future antimicrobial approaches.
